# An Unusual 2,3-Secotaraxerene and Other Cytotoxic Triterpenoids from *Pleiocarpa pycnantha* (Apocynaceae) Leaves Collected from Nigeria

**DOI:** 10.3390/molecules19033389

**Published:** 2014-03-20

**Authors:** Olubunmi A. Omoyeni, Mervin Meyer, Emmanuel Iwuoha, Ivan Green, Ahmed A. Hussein

**Affiliations:** 1 Department of Chemistry, University of the Western Cape, Private Bag X17, Bellville 7535, South Africa; 2 Department of Biotechnology, Apoptosis Research Unit, University of the Western Cape, Private Bag X17, Bellville 7535, South Africa

**Keywords:** *Pleiocarpa pycnantha*, Apocynaceae, triterpenoids, cytotoxicity, 2,3-*seco*-taraxerene, ursolic acid, WST-1

## Abstract

Three known triterpenoids, namely ursolic acid (**1**), and the 27-*E-* and 27-*Z-p*-coumaric esters of ursolic acid (compounds **2**, **3**), were isolated together with a new triterpene 2,3-*seco*-taraxer-14-en-2,3-lactone [pycanocarpine (**4**)] from an ethanolic extract of *Pleiocarpa pycnantha* leaves. The structure of **4** was unambiguously assigned using NMR, HREIMS and X-ray crystallography. The cytotoxic activities of the compounds were evaluated against HeLa, MCF-7, KMST-6 and HT-29 cells using the WST-1 assay. Ursolic acid (**1**) displayed potent cytotoxic activity against HeLa, HT-29 and MCF-7 cells with IC_50_ values of 10, 10 and 20 µM respectively. The new compound **4** and its hydrolysed derivative **5** were selectively cytotoxic to the breast cancer cell line, MCF-7 with IC_50_ values 20 and 10 µM respectively. This is the first report on isolation of a 2,3-*seco*-taraxerene derivative from the Apocynaceae family and cytotoxic activityof *P. pycnantha* constituents.

## 1. Introduction

Man has used plants for food and medicinal purposes for thousands of years and thereby has acquired detailed knowledge of their properties [1,2,3]. Medicinal plants have been in use to treat various diseases for many years in different parts of the world [4]. The use of natural products is also on the increase in the Western world [5]. These natural products are used either as standardized plant extract, semi purified or purified forms [6]. According to the World Health Organization [7], about 70%–95% of the world’s population in developing countries relies mainly on plants for their primary health care. Traditional medicine remains the only health resource available to about 60% of the world’s population, especially those in the vast rural areas of developing countries [8,9].

Cancer is a major public health burden in both developed and developing countries. According to the global cancer statistics made public by the American Cancer Society in 2007, the total number of cancer deaths per day was 20,000 with 38% in developed countries and 62% in developing countries. It was also projected that 27 million new cancer cases and 17.5 million cancer deaths would occur by 2050. Siegel *et al.* [10] performed one of the most recent studies on cancer incidence, mortality, and survival based on incidence from the National Cancer Institute, the Centers for Disease Control and Prevention, and the North American Association of Central Cancer Registries and mortality data from the National Center for Health Statistics. A total of 1,638,910 new cancer cases and 577,190 deaths from cancer were projected to occur in the United States in 2012. Plants have a long history of use in in cancer treatment [11]. Over 60% of currently used anticancer agents are derived from natural sources [12,13]. Traditionally, Apocynaceae plants were used to treat gastrointestinal ailments, fevers, malaria, pain, diabetes, ulcers, tuberculosis, helminthosis and cancer [14,15,16,17,18,19]. *Pleiocarpa pycnantha* is a shrub or small to large tree attaining of highest up to 30 m in secondary jungle or in the lower level of the high-forest from Mali to South Nigeria and across Africa to Zaire, Angola, Uganda and Zanzibar. The wood is hard and yellow, and it’s used to make combs, plane-blocks and sundry small objects. In Ghana, the roots are added to palm-wine to give it potency. Ground roots mixed with seeds of *Aframomum melegueta* K. Schum. and palm wine is taken as a laxative. In Benin, leaf maceration with lemon juice is given to patients suffering from jaundice, oedema, reduced urine excretion and infection by roundworms [20]. In the Yoruba speaking part of West Africa a blend of the leaves of *P. pycnantha*, *Spondias mombin* (Anacardiaceae) and a fruit of *Aframomum melegueta* (Zingiberaceae), are combined and administered to gain and retain good memory [21]. Some indole alkaloids e.g., pycnanthine, pleiocarpamine, quebrachamine macusine and (−)-ebunarmine have been isolated from *P. pycnantha* roots and bark [22,23]. Pleiocarpamine has demonstrated anticancer potential [24]. In this work, we explored the anticancer potential of *P. pycnantha*, its extracts and isolated compounds. To the best of our knowledge, this is the first substantial biological activity reported for *P. pycnantha* leaves and the first on isolation of triterpenes for the specie, and the second for the genus.

## 2. Results and Discussion

**Figure 1:**
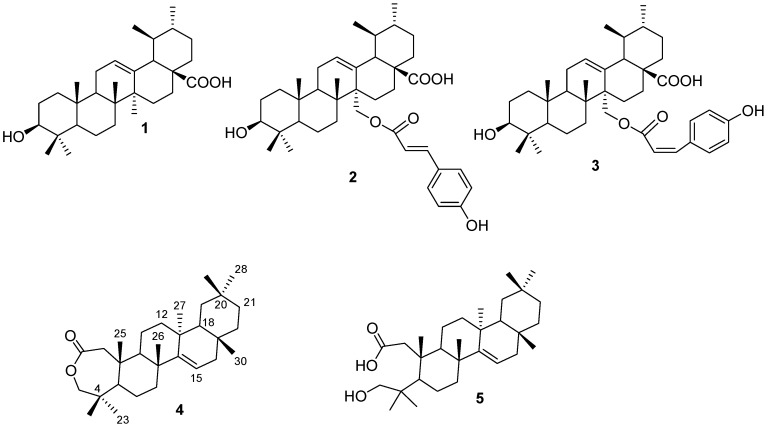
Chemical structures of isolated compounds from *P. pycnantha*.

An ethanol extract of *P. pycnantha* leaves was evaluated for cytotoxicity on cervical carcinoma (HeLa), breast adenocarcinoma (MCF-7), colorectal adenocarcinoma (HT-29) and non-cancerous fibroblast (KMST-6) human cell lines, the extract treatment induced loss of cell viability in a dose-dependent manner. However the activity was low with IC_50_ > 100 µg/mL on all the cell lines, some of the fractions demonstrated potent activity at <100 μg/mL, which gave some justification to carry out further chemical investigations of such fractions. The active fractions were further purified which led to the isolation of the active constituents **1**–**4** (Figure 1). Repeated chromatography of the fractions P4, P9 and P12 led to the isolation of three known triterpenoids; ursolic acid (**1**) [25,26], 27-*p*-*E*-coumaroloxyursolic acid (**2**) [27], 27-*p*-*Z*-coumaroyloxyursolic acid (**3**) [28], and a new triterpene, which we have named pycanocarpine (**4**), which was further hydrolyzed to give the corresponding 2-carboxy-3-hydroxy derivative **5**. The known compounds were identified by comparison of their NMR data with published values [25,26,27,28].

Triterpene esters containing the *E*-coumaric acid isomer have been reported from natural sources, while, the co-existence of *E*- and *Z*-coumaric acid esters of ursolic acid (compounds **2** and **3**) in *Plumeria obtusa* of the same family has been reported once [27,28]. However, although some reports have indicated the photo isomerization of the *E* isomers to the *Z* form using UV light [29], we did not observe any such change (as indicated by ^1^H-NMR measurements) in any of the isomers during 2–3 months under normal working conditions.

**Figure 2:**
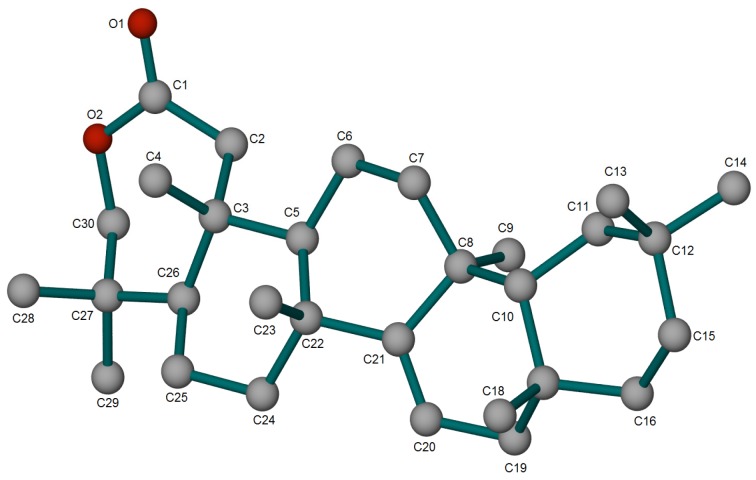
ORTEP diagram for **4**.

**Figure 3:**
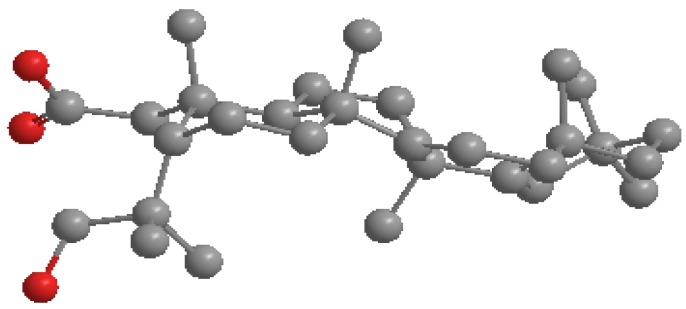
Ball and Stick minimized energy model for **5**.

Compound **4** was isolated as colourless needle-like crystals with molecular formula of C_30_H_48_O_2_ on the basis of TOF-ESI-MS *m*/*z* 441.3735[M+H]^+^. The IR spectra showed peaks at 1,738 cm^−1^ (C=O stretching) and 1,474 cm^−1^ (C-O). The NMR spectroscopic data of **4** are similar to those of 3β-taraxerol, with differences limited to ring A. The signal due to the olefinic proton at δ 5.54 (m) together with ^13^C-NMR signals of C-14 (δ 157.7) and C-15 (δ 117.1) suggested a taraxerane moiety [30]. The signal at δ 174.8 (C-2), 77.9 (C-3) in addition to oxygenated methylene protons at δ 3.79 and 4.08 (CH_2_-3) indicated the presence of a lactone group. The ring A of compound **4** showed some similarity when it was compared to 4-hydroxy-3,4-*seco*-ursan-12-en-28-oic acid 3,4 lactone derived from ursolic acid [31] and compared very favourably. The NMR data were assigned unambiguously from 2D NMR spectral analysis of compound **4** and its hydrolyzed product **5**. The structure was finally established from X-ray diffraction studies. Figure 2 illustrates the perspective view of the molecule with its relative configuration. The compound was thus assigned as 2,3-*seco*-taraxer-14-en-2,3-lactone and given the name pycanocarpine. After alkaline hydrolysis, compound **4** produced the unstable derivative *viz* 2,3-*seco*-3-hydroxy-taraxer-14-en-3-oic acid (**5**) as an off-white amorphous powder. The TOF-ESI-MS indicated an *m*/*z* 459.3889 [M+H]^+^. It was noticeable that the free carboxylic and hydroxyl groups would easily reform the lactone ring after a short time in organic solvents such as methanol and chloroform. The NMR data of **5** is similar to the parent compound **4** and is illustrated in Table 1. Minor changes were observed between **4** and **5** for carbons C-11, -23, -24, and -26. The stereochemistry of both compounds, especially ring A, may partially explain this. In **4** the carbonyl group is directed into the same plan similar to C-24, -26 and -11. On the other hand, in derivative **5**, the minimized energy conformation (Figure 3) showed different orientations and distances for the C_3_-OH and C_2_OOH groups.

**Table 1:** ¹H/¹³C-NMR data of 4 (CDCl_3_) and 5 (CDCl_3_+ 1drop CD_3_OD) (δ values, *J* in parenthesis in Hz).

Position	Compound 4		Compound 5	
	^13^C	^1^H (*J* Hz)	^13^C	^1^H (*J* Hz)
1	44.9 t	2.44 br s; 2.78 br s	43.1 t	2.36 d, 2.35 d, (12.0)
2	174.8 s	-----	172.9 s	-----
3	77.9 t	3.79 br s; 4.08 br s	72.1 t	3.33 d; 3.34 d, (7.0)
4	38.4 s	-----	38.6 s	-----
5	48.7 d	0.97 *	44.9 d	1.72 *
6	20.6 t	1.57 *; 1.66 *	21.8 t	1.60 *; 1.53 *
7	35.1 t	1.02 *; 1.35 *	35.6 t	0.95 *; 1.34 *
8	35.8 s	-----	34.9 s	-----
9	40.9 d	1.64 *	42.8 d	1.76 *
10	46.7 s	-----	43.6 s	-----
11	18.6 t	1.57 *; 1.75 br s	29.5 t	1.18 * (2H)
12	36.6 t	0.98 (dd; 12.0, 4.5); 1.34 *	37.5 t	1.49 *; 1.83 (td; 9.0, 3.5)
13	37.4 s	-----	37.0 s	-----
14	157.5 s	-----	157.3 s	-----
15	117.1 d	5.54 (dd; 8.0, 3.5)	116.8 d	5.46 dd, (9.0, 4.5)
16	37.8 t	1.64 *; 1.92 (dd; 9.0, 2.0)	36.5 t	0.90 *; 1.22 *
17	40.6 s	-----	40.2 s	-----
18	48.7 d	0.97 *	48.5 d	0.92 * (2H)
19	39.4 t	1.37 *; 2.04 (dt; 9.5, 3.5)	39.7 t	1.33*; 1.96 (dd; 3.5, 8.5)
20	28.7*s	-----	28.6 s	-----
21	33.1 t	1.25 (dt; 9.5, 3.2); 1.32 *	32.9 t	1.44 *; 1.59 *
22	33.6 t	1.60 *; 1.64 *	33.8 t	1.55 *; 1.60 *
23	19.4 q	1.02 s	26.3 s	0.93 s
24	28.7 q	0.95 s	24.4d	1.04 s
25	17.5 q	1.13 s	18.5 q	1.02 s
26	25.8 q	1.11 s	20.6 q	0.86 s
27	29.6 q	0.92 s	29.5 q	0.83 s
28	21.1 q	0.90 s	23.9 q	1.00 s
29	33.5 q	0.95 s	33.1 q	0.87 s
30	29.6 q	0.83 s	29.7 q	0.71 s

* overlapped signals.

**Figure 4:**
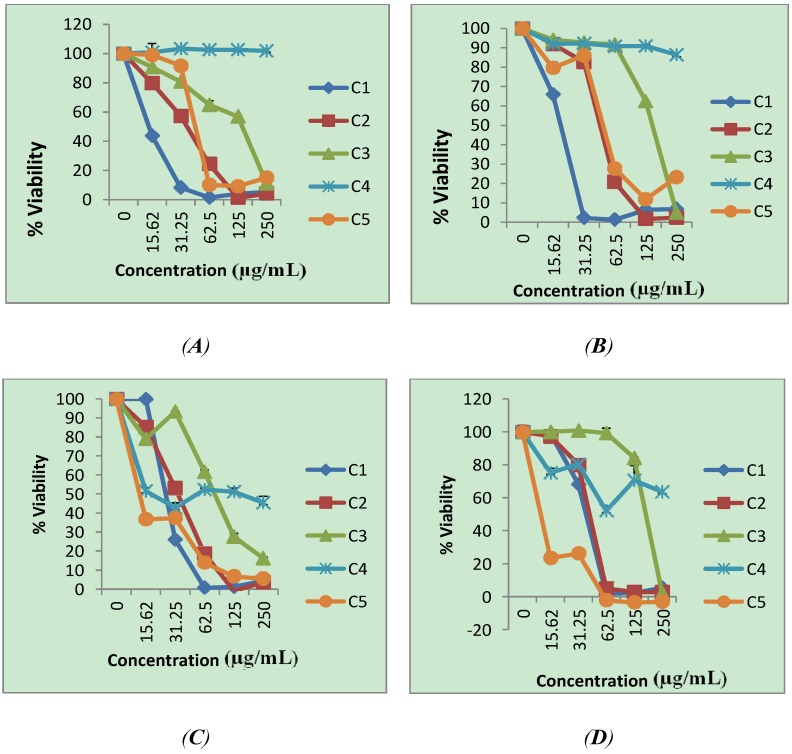
Effect of compounds **1**–**5** on cell viability of (**A**) HeLa cells (**B**) HT-29 (**C**) MCF-7 (**D**) KMST-6 in a concentration dependent manner as measured by WST-1 assay.

The five compounds **1**–**5** were examined for their dose-response effect on the viability of HeLa, HT-29, MCF-7 and KMST-6 cells using the WST-1 assay (Figure 4). All the compounds isolated from *P. pycnantha* leaves inhibited the growth of cancer cell on specific cell lines except **4** from fraction P4 which did not show cytotoxicity on HeLa and HT-29 cells at the dosages used in this study. The cytotoxicity activity ranged from low to moderate to high. The results from the WST-1 assay were used to determine the IC_50_ values for all the compounds on the four cell lines. Generally, the highest cytotoxicity was demonstrated by ursolic acid (**1**) with an IC_50_ value of 10 μM on HeLa and HT-29 cells and 20 μM on MCF-7 cells (Table 2). Compound **2** was moderately cytotoxic with IC_50_ values of 50, 100 and 60 μM on HeLa, MCF-7, and HT-29 respectively (Table 2). Compounds **3** and **4** were weakly cytotoxic on all cancer cell lines and non- cytotoxic to the non-cancerous cell line KMST-6. The IC_50_ value of **3** ranged from 180 μM to 300 μM on the cancer cells. The new compound **4** displayed cytotoxicity to the adenocarcinoma cell line, MCF-7 with an IC_50_ value of 20 μM while it was non cytotoxic on other cell lines, including the non-cancerous cell line KMST-6. The selectivity index (SI) for **4** was > 30 for MCF-7 cells, demonstrating that the activity of this compound is highly selective for MCF-7 cells. Compound **5** was also highly cytotoxic to MCF-7 cells and the non-cancerous KMST-6 cells with IC_50_ value of 10 μM. Compound **5** was only moderately cytotoxic on HeLa and HT-29 cells with IC_50_ values of 180 μM and 170 μM, respectively. Generally, the IC_50_ values for ursolic acid (**1**) on all cell lines tested in this study was lower compared to **2** and **3**. This is an indication that ursolic acid was more cytotoxic than its 27-ester derivatives. A similar trend was observed by Shao *et al.* [32] where it was suggested that the introduction of bulky groups containing benzene to the 3-OH position could increase the steric hindrance and decrease binding to the target.

**Table 2:** IC_50_ (μM) values for compounds **1**–**5** as determined by WST-1 assay.

Compound	Cell lines						
	HeLa		HT-29		MCF-7		KMST-6
	IC_50_	SI	IC_50_	SI	IC_50_	SI	IC_50_
**1**	10	10.0	10	10.0	20	5.0	100
**2**	50	2	100	1	60	1.7	100
**3**	200	1.5	230	1.3	180	1.7	300
**4**	>600	-----	>600	-----	20	>30	>600
**5**	180	0.06	170	0.06	10	1	10

Selectivity Index (SI) = IC_50_(KMST-6)/IC_50_(HeLa) or any of the cancer cell lines used.

Cancer being the second largest disease makes a sizeable contribution to the total number of deaths. The World Cancer Report documented that cancer rates are set to increase globally at an alarming rate [33]. Plants usage has been the basis of prevention and treatment of diseases for centuries and it is thus not surprising that development of anticancer drugs today started from their preliminary cytotoxicity screening. Ursolic acid (**1**) is an ursane type triterpene found mainly in the leaves and displays several important biological activities *viz*: anti-inflammatory, antioxidant and anti-cancer properties [34]. Compound **3** has been reported to have potent activity against acetyl CoA cholestryl acyl tranferase (ACAT) [35]. The anti-proliferative activity of ursolic acid (**1**) has been reported in a wide variety of cancer cell lines [36]. Ursolic acid hydroxycinnamate esters isolated from cranberry fruit has been evaluated for anti-tumour activity in a 60 tumour cell line panel through the National Cancer Institute’s Developmental Therapeutics program. The research showed that the esters inhibited the growth of lung, colon, breast, renal and leukemia cancer cell lines with GI_50_ ranging from 1.2–11 µM based on sulforhodamine B(SRB) assay [37]. The *cis*- and *trans*- isomers of 3-*O*-p-hydroxycinnamoyl ursolic acid has been reported to inhibit tumour growth *in vitro* with the *cis*-3-*O*-p-hydroxycinnamoyl ursolic acid showing slightly greater activity in most cell lines with GI_50_ values ~20 µM in MCF-7 breast, ME 180 cervical, and PC3 prostate tumour cell lines [38]. The anti-proliferative activity hydroxycoumaroyl esters of ursolic acids were reported on A-549, HCT-15, MCF-7, HT-1197 with IC_50_ ranging from 0.5 to 6.5 µg/mL, from *Uncaria rhynchophylla* [39]. Many plants from Apocynaceae family have been reported for anticancer activity [40], including this report, which indicated the importance of their constituents as an interesting source for the discovery of new anticancer agents.

## 3. Experimental

### 3.1. General

NMR Spectra were measured on 200 Gemini-Varian (Varian Associates Inc., Palo Alto, CA, USA) and 400 MHz Bruker (Bruker BioSpin Corporation, Billerica, MA, USA) NMR spectrometers and the chemical shifts were expressed in ppm relative to CDCl_3_ signal (7.24 ppm for ^1^H, and 77.00 ppm for ^13^C). TOF-ESI-MS spectra were measured using a HP-5 ms (30 m × 0.25 mm ID, 0.25 µm film thickness) column on a Waters GCT system (Waters Corporation, Milford, MI, USA) equipped with a CTC CombiPAL Autosampler (Agilent Technologies, Santa Clara, CA, USA). IR spectra were recorded on a Perkin Elmer Spectrum 400 spectrometer (PerkinElmer, MA, USA). An Agilent 1100 HPLC system (Agilent Technologies, Santa Clara, CA, USA) consisting of a quaternary solvent delivery system, an on-line degasser, an auto-sampler, a column temperature controller and ultraviolet detector coupled with an analytical workstation and Discovery^®^ C18 column, 5 μm, 250 mm × 10 mm i.d. (Sigma-Aldrich, St. Louis, MO, USA). TLC was performed on normal phase Merck Silica gel 60 F_254_ on pre-coated aluminium plates (0.2 mm; Merck, Dermstadt, Germany). Column chromatography was carried out using 60 Å silica gel (230–400 mesh; Merck).

### 3.2. Plant Material

*Pleiocapa pycnantha* (K. Schum.) Stapf was collected in Ikere Ekiti, Ekiti State, Nigeria in December, 2010. The botanical identification was done by Femi Omotayo of the Herbarium section of Plant Science Department of Ekiti State University, Ado-Ekiti, Nigeria, where a voucher specimen was deposited.

### 3.3. Extraction and Isolation

The ground air-dried leaves (~1.0 kg) were soaked in 95% ethanol for three days and the extract then filtered and concentrated in vacuum at ~ 40 °C to yield 81.0 g of the extract. Part (62.0 g) of the total extract was applied on silica gel column and eluted with a mixture of hexane-EtOAC of increasing polarity. Fractions showing similar TLC characteristics were pooled together and concentrated in vacuum, giving rise to 13 fractions coded P1–P13 that were screened for cytotoxicity using WST-1 assay. Fraction P4, P7, P8 and P9 displayed strong cytotoxicity while P12 was moderately cytotoxic and were submitted for further purification. P9 (4.0 g) was chromatographed on a silica gel column using gradient elution with EtOAc/hexane (20:80–100:0) to afford Q1-13; Q4 (310 mg) was crystallized from 10% hexane/EtOAc to give compound **1** (100 mg). P12 (5.2 g) was chromatographed on silica gel column using EtOAc/hexane (50:50–100:0) to afford sub-fraction A-H. P12E (140 mg) was further purified on sephadex LH-20 column using DCM/MeOH(95:5) and HPLC (MeOH/H_2_O,80:20) to afford compound **2** (5.5 mg) and **3** (7.3 mg)**.** Fraction P4 (6.0 g) was chromatographed on silica gel using EtOAc/hexane (2:98–100:0) to give T1–T10, sub-fraction T6 was recrystallized from hexane to produce compound **4** (0.97g). Compound **5** was synthesized by refluxing 33.0 mg of **4** in methanolic/KOH (5.0%) for 3 h to afford R1 mixture which was purified on silica gel column using hexane/EtOAC (70:30) to yield 20 mg (60%).

### 3.4. Spectra Data

*Pycanocarpine* (**4**). Needle-like crystals, mp 246–249 °C; IR (KBr) V_max_ 3054, 2914, 2856, 1738, 1474, 1077 cm^−1^. TOF-ESI-MS *m/z* 441.3735 [M+H]^+^ (calcd 440.3654 for C_30_H_48_O_2_); ^1^H and ^13^C-NMR data see Table 1.

*2,3-Seco-3-hydroxy-taraxer-14-en-3-oic acid* (**5**); Off-white amorphous powder, mp 237–241 °C; IR (KBr) *V*_max_ 3055, 2921, 2857, 1740, 1473, 1067cm^−1^; TOF-ESI-MS *m*/*z* 459.3889 [M+H]^+^ (calcd 458.3760 for C_30_H_50_O_3_); ^1^H and ^13^C-NMR data see Table 1.

### 3.5. X-ray Structure Determination of **4**

Single-crystal X-ray diffraction data [41] were collected on a Bruker KAPPA APEX II DUO diffractometer (Bruker BioSpin Corporation, Billerica, MA, USA)) using graphite-monochromated Mo-Kα radiation (χ = 0.71073 Å). Data collection was carried out at 173(2) K. Temperature was controlled by an Oxford Cryostream cooling system (Oxford cryosystems Ltd, Oxford, UK). Cell refinement and data reduction were performed using the program SAINT [42]. The data were scaled and absorption correction performed using SADABS [43].

The structure was solved by direct methods using SHELXS-97 and refined by full-matrix least-squares methods based on F^2^ using SHELXL-97 [43] and using the graphics interface program X-Seed [44,45]. The programs X-Seed and POV-Ray [46] were both used to prepare molecular graphic images. All non-hydrogen atoms were refined anisotropically. All hydrogen atoms were placed in idealised positions and refined in riding models with U_iso_ assigned the values to be 1.2 or 1.5 times those of their parent atoms and the constraint distances of C-H ranging from 0.95 Å to 1.00 Å. The structure was refined to R factor of 0.0483. The Flack × parameter is equal to −0.0123 with 1.3609.

### 3.6. Culture of Cell Lines

HeLa, HT-29, MCF-7 and KMST-6 cells were prepared from our laboratory stocks. Briefly, cell monolayers were maintained in Dulbecco’s modified Eagle’s (DMEM, Lonza Group Ltd., Base, Switzerland) medium with phenol red supplemented with 10% foetal bovine serum (FBS, Biochrom AG, Berlin, Germany) and 1% Penicillin–streptomycin (Lonza Group Ltd.). All cells were maintained in a humidified incubated at 37 °C in an atmosphere of 5% CO_2_. The cells were harvested using trypsin (Lonza Group Ltd.), and viable cell concentrations were determined using the Countess^®^ Automated Cell Counter (Invitrogen, CA, USA). 5 × 10^4^ viable cells were added to each well of a 96-well tissue culture plate and incubated overnight at 37 °C under 5% CO_2_ in a humidified incubator to allow cells to attach to wells.

### 3.7. WST-1 Based Cytotoxicity Assay

Cell viability was measured using the WST-1 assay (Roche Diagnostics GmbH, Mannheim, Germany) as previously described by Ngamwongsatit *et al.*, 2008. Cells were plated in 96-well cell culture plates at a concentration of 5 × 10^4^ cells/well in 100 µL culture medium. After 24 h the cells were treated with increasing concentrations of ethanol extract (0–2 mg/mL), fractions (0–2 mg/mL) and purified compounds (0–250 µg/mL) for a further 24 h. The extracts, fractions and compounds were prepared in DMSO and then mixed with the culture medium. The final concentrations of the DMSO were less than 0.1%. Following 24 h treatment, 10 µL of cell proliferation reagent WST-1 was added and the plates were incubated for 4 h at 37 °C under 5% CO_2_ in a humidified incubator. The plates wereshakenfor 1 min on a shaker and the absorbance of the samples were measured at 450 nm (reference wavelength was 750 nm) on a micro-plate reader. The cell viability was calculated using the following formula:

% Viability = OD_treated well_ − OD_blank_/OD_untreated well_ − OD_blank_ (1)

IC_50_ values were calculated using Prism Graph pad software. Triplicate experiments were conducted and the results expressed as mean ± SEM.

## 4. Conclusions

The phytochemical study of the ethanolic extract from the leaves from *Pleiocarpa pycnantha* has led to the isolation a new traxerane-type triterpenoid, in addition to three known compounds. 2,3-*Seco* triterpenoids are rare in Nature and compound **4** represents the first example of this type to be isolated from the Apocynaceae family. This is the first report of the isolation of triterpenes from the plant and to the best of our knowledge, the first significant bioactivity report. Our studies suggested that pure compounds isolated from *Pleiocarpa pycnantha* demonstrate cytotoxic activity against cervical, breast and colon cancer.

## References

[1] Trease G.E., Evans W.C. (1996). Pharmacognosy.

[2] Samuelsson G. (2004). Drugs of Natural Origin. A Textbook of Pharmacognosy.

[3] Brouwer N., Liu Q., Harrington D., Kohen J., Vemulpad S., Jamie J., Randall M., Randall D. (2005). An ethnopharmacological study of medicinal plants in New South Wales. Molecules.

[4] Adebayo J.O., Krettli A.U. (2011). Potential antimalarials from Nigerian plants: A review. J. Ethnopharmacol..

[5] Helms S. (2004). Cancer prevention and therapeutics: *Panax ginseng*. Altern. Med. Rev..

[6] Cos P., Vlietinck A.J., Berghe D.V., Maes L. (2006). Anti-infective potential of natural products: How to develop a stronger *in vitro* ‘proof-of-concept’. J. Ethnopharmacol..

[7] World Health Organization (WHO) Traditional Medicine. http://www.who.int/topics/traditional_medicine/en/.

[8] Le Grand A., Wondergem P. (1989). Herbal Medicine and Health Promotion. A Comparative Study of Herbal Drugs in Primary Health Care.

[9] Cordell G.A. (1995). Changing strategies in natural products chemistry. Phytochemistry.

[10] Siegel R., Naishadham D., Jemal A. (2012). Cancer Statistics 2012. Cancer J. Clin..

[11] Hartwell J.L. (1982). Plants Used Against Cancer.

[12] Cragg G.M., Kingston D.G.I., Newman D.J. (2005). Anticancer Agents from Natural Products.

[13] Newman D.J., Cragg G.M., Snader K.M. (2003). Natural products as sources of new drugs over the period 1981–2002. J. Nat. Prod..

[14] Wiart C. (2006). Medicinal Plants of Asia and the Pacific.

[15] Gan L.S., Yang S.P., Wu Y., Ding J., Yue J.M. (2006). Terpenoid indole alkaloids from *Winchia calophylla*. J. Nat. Prod..

[16] Lim S.H., Sim K.M., Abdullah Z., Hiraku O., Hayashi M., Komiyama K., Kam T.S. (2007). Leuconoxine, kopsinitarine, kopsijasmine, and kopsinone derivatives from Kopsia. J. Nat. Prod..

[17] Wang G.C., Zhong X.Z., Zhang D.M. (2011). Two pairs of epimeric indole alkaloids from *Catharanthus roseus*. Planta Med..

[18] Macabeo A.P.G., Vidar W.S., Chen X., Decker M., Wan J.H.B., Franzblau S.G., Galvez E.V., Aguinaldo M.A.M., Cordell G.A. (2011). Mycobacterium tuberculosis and cholinesterase inhibitors from *Voacanga globosa*. Eur. J. Med. Chem..

[19] Aremu A.O., Finnie J.F., van Staden J. (2012). Potential of South African medicinal plants used as anthelmintics—Their efficacy, safety concerns and reappraisal of current screening methods. S. Afr. J. Bot..

[20] Burkill H.M. (1985). The Useful Plants of West Tropical Africa 2nd Edition Volume 1, Families A–D.

[21] Fatumbi V.P. (1995). Ewé: The Use of Plants in Yoruba Society.

[22] Gorman A.A., Schmid H. (1967). Structure of dimeric indole alkaloid pycnanthinine. Monatsh. Chem..

[23] Gorman A.A., Dastoor N.J., Manfred H., von Philipsborn W., Renner U., Schmid H. (1969). Alkaloids. CXXXII. Constitution of two new type dimeric indole alkaloids, pycnanthine and pleiomutinine. Helv. Chim. Acta.

[24] Keawpradub N., Houghton P.J., Eno-Amooquaye E., Burke P.J. (1997). Activity of extracts and alkaloids of Thai Alstonia species against human lung cancer cell lines. Planta Med..

[25] Seebacher W., Simic N., Weis R., Saf R., Kunert O. (2003). Complete assignments of ^1^H and ^13^C-NMR resonances of oleanolic acid, 18α-oleanolic acid, ursolic acid and their 11-oxo derivatives. Magn. Reson. Chem..

[26] Fontanay S., Grare M., Mayer J., Finance C., Duval R.E. (2008). Ursolic, oleanolic and betulinic acids: Antibacterial spectra and selectivity indexes. J. Ethnopharmacol..

[27] Siddiqui S., Siddiqui B.S., Naeed A., Begum S. (1990). Three pentacyclic triterpenoids from the leaves of *Plumeria obtusa*. J. Nat. Prod..

[28] Budzikiewicz H., Thomas H. (1980). p-Cumaroxy-ursolsaure, ein neuer inhaltstoff von Ilex aquifolium. L. Z. Naturforsch..

[29] Danylec B., Iskander M. (2002). ^1^H-NMR measurement of the *trans*-*cis* photoisomerization of cinnamic acid derivatives. J. Chem. Ed..

[30] Mahato S.B., Kundu A.P. (1994). ^13^C-NMR Spectra of pentacyclic triterpenoids—A complication and some salient features. Phytochemistry.

[31] Tu H.Y., Huang A.M., Wei B.L., Gan K.H., Hour T.C., Pu Y.S., Lin C.N. (2009). Ursolic acid derivatives induce cell cycle arrest and apoptosis in NTUB1 cells associated with reactive oxygen species. Bioorg. Med. Chem..

[32] Shao J.W., Dai Y.C., Xue J.P., Wang J.C., Lin F.P., Guo Y.H. (2011). *In vitro* and *in vivo* anticancer activity evaluation of ursolic acid derivatives. Eur. J. Med. Chem..

[33] World Health Organization (WHO) Media Centre. http://www.who.int/mediacentre/news/releases/2003/pr27/en/.

[34] Silva J.A., Silva A.G., Alves A.S., Reis R., Nascimento C.C., Diré G.F., Barreto A.S. (2013). *Plumeria rubra* (Apocynaceae): A good source of ursolic acid. J. Med. Plants Res..

[35] Nishimura K., Fukuda T., Miyase T., Noguchi H., Chen X.M. (1999). Activity-guided isolation of triterpenoid acyl CoA cholesteryl acyl transferase (ACAT) inhibitors from *Ilex kudincha*. J. Nat. Prod..

[36] Neto C.C. (2007). Cranberry and its phytochemicals: A review of *in vitro* anticancer studies. J. Nutr..

[37] Kondo M. (2006). Phytochemical Studies of Extracts from Cranberry (*Vaccinium macrocarpon*) with Anti-Cancer, Anti-Fungal and Cardioprotective Properties. M.S. Thesis.

[38] Hamzah A.S., Lajis N.H. (1998). Chemical constituents of *Hedyotis herbacea*. ARBEC.

[39] Lee J.S., Kim J., Kim B.Y., Lee H.S., Ahn J.S., Chang Y.S. (2000). Inhibition of Phospholipase Cγ1 and Cancer Cell Proliferation by Triterpene Esters from *Uncaria rhynchophylla*. J. Nat. Prod..

[40] Okouneva T., Hill B.T., Wilson L., Jordan M.A. (2003). The effects of vinflunine, vinorelbine, and vinblastine on centromere dynamics. Mol. Cancer Ther..

[B41-molecules-19-03389] 41.Crystallographic data (excluding structure factors) for the structures reported in this paper have been deposited with the Cambridge Crystallographic Data Centre as supplementary crystallographic data (CCDC 972299) for **4** and can be obtained free of charge via www.ccdc.cam.ac.uk/conts/retrieving.html (or from the Cambridge Crystallographic Data Centre, 12 Union Road, Cambridge CB21EZ, UK; fax: (+44) 1223-336-033; or deposit@ccdc.cam.ac.uk).

[42] Bruker (2006). SAINT Version 7.60a.

[43] Sheldrick G.M. (1997). SHELXS-97, SHELXL-97 and SADABS Version 2.05.

[44] Barbour L.J. (2001). X-Seed—A Software Tool for Supramolecular Crystallography. J. Supramol. Chem..

[45] Atwood J.L., Barbour L.J. (2003). Molecular Graphics: From Science to Art. Cryst. Growth Des..

[46] POV-Ray for Windows (Version 3.6). http://www.povray.org.

